# Fine-scale linkage disequilibrium mapping: a comparison of coalescent-based and haplotype-clustering-based methods

**DOI:** 10.1186/1753-6561-1-s1-s133

**Published:** 2007-12-18

**Authors:** Sungho Won, Ritwik Sinha, Yuqun Luo

**Affiliations:** 1 Department of Epidemiology and Biostatistics, Case Western Reserve University, Wolstein Research Building, 10900 Euclid Avenue, Cleveland, Ohio 44106, USA

## Abstract

Among the various linkage-disequilibrium (LD) fine-mapping methods, two broad classes have received considerable development recently: those based on coalescent theory and those based on haplotype clustering. Using Genetic Analysis Workshop 15 Problem 3 simulated data, the ability of these two classes to localize the causal variation were compared. Our results suggest that a haplotype-clustering-based approach performs favorably, while at the same time requires much less computing than coalescent-based approaches. Further, we observe that 1) when marker density is low, a set of equally spaced single-nucleotide polymorphisms (SNPs) provides better localization than a set of tagging SNPs of equal number; 2) denser sets of SNPs generally lead to better localization, but the benefit diminishes beyond a certain density; 3) larger sample size may do more harm than good when poor selection of markers results in biased LD patterns around the disease locus. These results are explained by how well the set of selected markers jointly approximates the expected LD pattern around a disease locus.

## Background

Among the various linkage disequilibrium (LD) fine-mapping methods, two broad classes have received considerable development recently: those based on coalescent theory and those based on haplotype clustering. Three particular implementations seem promising: TreeLD by Zollner and Pritchard [1], the software by Molitor et al. [2], and that by Waldron et al. [3], the first one based on coalescent theory and the latter two (referred to as MOL and WAL) based on haplotype clustering.

Coalescent-based LD fine mapping explicitly models the history of current genetic variations. However, this conceptual advantage poses serious challenges to implementation due to the large number of parameters required to specify evolutionary details and the computational demand. TreeLD considers a series of focal points within the chromosomal region of interest. At each focal point, local approximation to the ancestral recombination graph that traces the mutation and recombination events backward in time is implemented. Next, the likelihood ratio at each focal point is computed and the inference on the location of the disease mutation is based on the likelihood ratios. Haplotype-clustering-based LD fine mapping, on the other hand, attempts to capture the population genetic principles via a distance measure between haplotypes, which will guide the spatial clustering of current haplotypes. The computing demand is much more manageable and hence larger data sets may be analyzed. A Bayesian partition model with a haplotype-based clustering algorithm was proposed by Molitor et al. [2]. However, the similarity measure used was not optimal in taking mutations and gene conversions into account. Under a similar framework, Waldron et al. [3] implements a novel similarity measure that takes into account allele frequencies and occasional mismatches from mutations or gene conversions.

Employing the simulated dense single-nucleotide polymorphism (SNP) data from Problem 3 of Genetic Analysis Workshop 15 (GAW15) and knowing the "answer", we compared the ability of the three approaches (TreeLD, MOL, and WAL) to localize the causal SNP, based on the root mean squared errors (RMSE) of the estimates of the causal SNP location, and the empirical coverage and the precision of the confidence/credible intervals of the location. In addition, effects of various factors were investigated: 1) choice of SNPs (tagging or equally spaced), 2) sample size, and 3) marker density.

## Methods

We considered the binary trait rheumatoid arthritis (RA), where the causal SNP location, the *HLA-DRB1 *locus on chromosome 6, is known. True haplotypes of each individual are assumed to be known. Within each replicate, a case-control sample with equal numbers of cases and controls is constructed by randomly selecting one individual from each affected sibling pair as a case. Attention is restricted to within 1 cM around the DRB1 locus. Unless otherwise noted, we consider 50 cases and 50 controls from each replicate, with 50 equally-spaced (ES) SNPs around the causal SNP. All 100 replicates are used unless otherwise indicated. The following quantities are evaluated and compared: 1) RMSEs, in the unit of base pairs (bp), of the causal location estimates; 2) the average length of credible/confidence intervals across replicates (LCI); and 3) the empirical coverage of the disease locus by these intervals (ECR).

In the first experiment, the RMSE of the estimate of the location of the causal SNP was computed for each of the three approaches (TreeLD, MOL, and WAL) based on 20 replicates, due to the substantial computational demand of TreeLD. The two best methods, TreeLD and WAL, were then applied to compute the 95% confidence intervals.

The first analysis showed that WAL is preferable. Thus, the rest of the analyses involve only WAL. Next, we investigated the effects of various factors on the above three criteria. The first factor considered was the choice of SNPs. We excluded the causal SNP from the data, and selected tagging SNPs based on several *r*^2 ^thresholds (0.2, 0.4, 0.6, and 0.8). For comparison, sets of SNPs that are roughly equally spaced and similar in numbers to the tagging SNP sets were selected. The second factor we examined was the effects of sample size and marker density. A larger data set always contained a smaller data set to obtain reliable results.

## Results

### Coalescent-based versus haplotype-clustering-based methods

The RMSEs of the estimates of the causal SNP location, in the scale of 10^4 ^bp, was 6.8 for WAL, 9.3 for TreeLD, and 32.3 for MOL. The improvement of WAL relative to MOL is expected because of its improvement on the similarity measure. RMSE is only one possible measure of the localization performance. Confidence/credible intervals (CI) provide fuller information and were computed for TreeLD and WAL. The results are shown in Table 1. Mutation rate is unknown and we examined several plausible values as recommend by Zollner and Pritchard [1]. Both programs were run for a set of 52 tagging SNPs (*r*^2 ^cutoff of 0.2), which happens to exclude the causal SNP, and a set of 50 ES SNPs, which happens to include the causal SNP. TreeLD is liberal with correspondingly shorter CIs, while WAL was able to maintain the nominal coverage. The empirical coverage of TreeLD intervals increases with the mutation rate when tagging SNPs are used, whereas it is relatively constant, albeit liberal, when ES SNPs are used. The ES SNP set also provides more precise intervals (380 kb versus 570 kb) when WAL is applied. These two observations might be an artifact of the causal SNP being included in the set of ES SNPs but not in the set of tagging SNPs. We will come back to the comparison of choice of SNPs later.

**Table 1:** Properties of 95% confidence intervals obtained from TreeLD and WAL

	TreeLD mutation rates^b,c^					
		
Choice of SNPs^a^	0.2	0.4	0.6	0.8	1.0	WAL^b,d^
Tagging SNPs	35% (2.9)	37% (2.8)	45% (3.3)	55% (3.7)	60% (3.8)	96% (5.7)
ES SNPs	65% (2.0)	59% (2.2)	59% (2.3)	59% (2.3)	65% (2.3)	96% (3.8)

^a^Either 50 equally spaced (ES) SNPs (causal locus included) or 52 tagging SNPs (causal locus missing) are used.

^b^Empirical coverage of the disease locus (entries outside the parentheses) and the average length of the intervals (entries inside the parentheses, in 10^5 ^bp) are given.

^c^TreeLD was applied to 20 replicates.

^d^WAL was applied to 100 replicates.

To perform a fair comparison, we increased the nominal coverage of TreeLD CIs until an empirical coverage of 95% is obtained, using the set of ES SNPs. This results in a nominal coverage of 99.9% with average length of intervals being 410 kb, compared to 380 kb of WAL intervals. Further, nominal 67% WAL intervals result in an empirical coverage of 65% and average length of 170 kb, compared to 200 kb for TreeLD intervals of the same empirical coverage. Thus, held to the same empirical coverage, TreeLD and WAL provide roughly the same precision of localization. However, WAL is much more stable in maintaining the nominal coverage. This, together with its computational advantage and ability to handle much larger data sets, renders the haplotype-clustering-based framework, as implemented in WAL, a clear winner in this situation.

### Choice of SNPs: tagging versus equally-spaced SNPs

There are a total of 219 SNPs within the 2-cM region considered, approximately one SNP per 9 kb. Using *r*^2 ^cutoff of 0.2, 0.4, 0.6, and 0.8, we obtained 52, 109, 141, and 178 tagging SNPs, respectively. We constructed four sets of ES SNPs, each with the same number of SNPs as one set of the tagging SNPs. WAL was run on the 100 replicates for each set of SNPs. Table 2 provides the three criteria: RMSE, LCI, and ECR of 95% intervals. For each SNP choice, the RMSE of the estimates and the precision of the intervals generally improve with increasing marker density. The empirical coverage was roughly maintained at the nominal level. For the same number of markers, the ES SNP set generally yields smaller RMSE and always provides more precise intervals than the corresponding set of tagging SNPs, though the difference become less pronounced with denser markers.

**Table 2:** Effects of choice of SNPs on estimates of causal SNP location

	Tagging SNPs			Equally spaced SNPs		
		
No. SNPs	RMSE (10^4 ^bp)	ECR	LCI (10^5 ^bp)	RMSE (10^4 ^bp)	ECR	LCI (10^5 ^bp)
52	12.5	96%	5.6	8.4	99%	3.7
109	6.9	97%	4.1	7.7	99%	3.5
141	10.1	93%	3.6	7.3	97%	3.2
178	5.9	100%	3.7	6.0	98%	3.1

**Figure 1:**
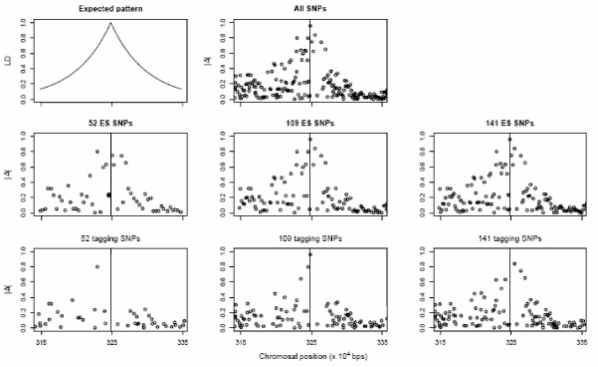
**LD patterns between the causal SNP and various sets of markers**. LD is measured with correlation (|Δ|) and is plotted against the physical distance between the causal SNP and other SNPs. The causal SNP is indicated with the vertical line within each plot.

Tagging SNPs are a rational choice for whole-genome or candidate-gene association studies. The above results seem to be counter-intuitive. However, our analyses focused on estimating the location of the causal variation, rather than detecting significant association, when there is already strong evidence that a causal variation exists within the region. Under certain assumptions, the LD between the causal SNP and a marker will decrease as the recombination fraction between them increases, and the relation between LD and physical distance (assuming it is proportional to recombination fraction) should follow the pattern displayed on the top left corner of Figure 1 after many generations. It is this pattern that provides information for fine-scale localization. To explore the reason of the seemingly counter-intuitive results, in Figure 1 we plot the absolute correlation coefficient (|Δ|) between the causal SNP and the members of the various SNP sets versus the physical distance between them. The totality of the SNPs within the 2-cM target region exhibits the expected LD pattern (top right plot). However, it is evident that not all markers are equally informative. Thus, it is foreseeable that if the LD pattern from a selected SNP set happens to deviate substantially from the expected one, either having a biased peak or substantial noise, poorer localization will result. Obviously, the set of 52 ES SNPs exhibits a pattern much closer to the expected one than that from the set of 52 tagging SNPs. This might explain the better localization ability of the ES SNPs when the markers are not too dense. As the density of the markers increases, there is substantial overlapping between the ES and tagging SNP sets and comparable localization ability results. To explore further, we removed the solid point (25 kb from causal SNP, |Δ| = 0.23) from the set of 52 ES SNPs, whose presence only obscures the LD pattern. This results in a 23% reduction in the MSE, through the reduction in the variance of the location estimates.

### Effect of marker density and sample size

To further understand the effect of marker density on the localization ability, we fixed the sample size at 100 individuals, and computed the three criteria for ES SNP sets of numbers ranging from 25 to 219, at an increment of 25, with all sets containing the causal SNP. Increasing the number of SNPs from 25 to 50 brings 56% reduction in MSE and 26% reduction in mean interval length. The improvement levels off as more markers are added until there are 125 markers in the set, at which point the MSE actually increases slightly with increasing density. However, the average interval length seems to remain quite constant, though without holding the empirical coverage constant (it fluctuates between 93% and 100%) no conclusions can be drawn regarding the intervals. These observations agree with our explanation above. Markers should be dense enough to reveal the LD pattern around the causal SNP, but not so dense that they increase the level of noise. On the other hand, in reality, causal variation is unknown and increased density improves the chance of capturing the causal SNP and thus can sharpen the LD pattern to improve the localization.

In a well designed study, increased sample size generally improves estimation. However, as seen here, in the problem of fine-scale localization, the fidelity of LD pattern revealed by the set of selected markers determines the localization ability. If the LD pattern of the set of selected markers is biased, a larger sample size will only drive the estimation closer to the biased peak. Using a set of 50 ES SNPs containing the causal SNP, we applied WAL to samples of sizes ranging from 50 to 400, at an increment of 50. The MSE, though fluctuating, exhibits an upward trend with increasing sample size. Decomposing the MSEs into the square of biases and variances, we observe that the bias shows an upward trend and the variance a downward trend. Though the causal SNP is included in this experiment, the totality of the SNPs jointly presents a biased LD pattern. The average length of 95% credible intervals and the empirical coverage both decrease with increasing sample size, the former from 580 kb to 177 kb and the latter from 100% to 66% when sample size goes from 50 to 400. The reduction in the variance component of the MSE brings the reduction in the interval length, whereas the increased bias brings the reduced empirical coverage.

## Discussion

In this article, we compare LD fine-mapping based on coalescent theory and haplotype clustering, as implemented in TreeLD, MOL, and WAL. In general, the best information that can be drawn for LD fine-mapping is the full coalescent genealogical events in the sample. TreeLD aims to approximate this information accurately within computational feasibility. In its current implementation, the upper limit is 85 SNPs genotyped on 440 chromosomes (220 individuals) [1]. On the contrary, MOL and WAL attempt to capture the recombination and mutation events into their similarity scores and thus reduce the computational demand substantially. Thus, much larger sets of SNPs can be analyzed. Using the GAW15 Problem 3 data, WAL performs best in terms of maintaining the nominal coverage and achieving the same precision as TreeLD with much less computation. TreeLD is liberal and computationally too intensive. MOL performs worst among the three competing methods, due to its similarity measure not appropriately accounting for mutation events. Thus, haplotype-clustering-based fine-scale localization methods, in the form implemented in WAL, are preferable among the three methods considered here. However, the generalization of this conclusion awaits more extensive simulation and applications.

Applying WAL, we examined the effects on localization performance of several factors. Several interesting results, some counter-intuitive, were observed: 1) when marker density is low (around 1 SNP per 38 kb), ES SNPs can improve the localization precision by as much as 34%, as compared to equal number of tagging SNPs; 2) increased marker density beyond a certain point (1 SNP per 16 kb), will not increase localization precision, and may result in higher RMSEs; 3) using a fixed set of SNPs, increasing the sample size may increase the RMSE and reduce the empirical coverage of CIs, even when the causal SNP is included. Under certain assumptions, the LD around the causal variation should follow a triangle pattern when plotted against the physical distance. How well the totality of the markers approximates this pattern determines the localization performance. A large sample size cannot compensate for poor selection of markers: a larger sample will actually drive the result toward the inherently biased location. Thus, judicious selection of SNPs that better reveal the true LD pattern is of ultimate importance. When constrained to have a low marker density, an equally spaced set of SNPs is preferred to tagging SNPs. What density will be considered low, however, depends on the disease and the locus under investigation. The empirical results here reflect the nature of RA and the *HLA-DRB1 *locus, where the signal is very strong.

## Conclusion

Using the GAW15 Problem 3 data, we demonstrate that the current implementation of haplotype-clustering-based fine mapping yields similar precision as coalescent-based approach, while being better in maintaining the nominal coverage and amenable to analyze larger data set. Judicious selection of SNPs in a putative region to capture the true LD pattern is crucial: equally spaced SNP sets are preferable to an equal number of tagging SNPs when marker density is low. Large sample size cannot compensate for poor selection of SNP sets.

## Competing interests

The author(s) declare that they have no competing interests.
